# Exosomal miRNA Changes Associated with Restoration to Sinus Rhythm in Atrial Fibrillation Patients

**DOI:** 10.3390/ijms25073861

**Published:** 2024-03-29

**Authors:** Pei-Chien Tsai, Albert Min-Shan Ko, Yu-Lin Chen, Cheng-Hsun Chiu, Yung-Hsin Yeh, Feng-Chun Tsai

**Affiliations:** 1 Department of Biomedical Sciences, Chang Gung University, Taoyuan City 33302, Taiwan; pctsai@gap.cgu.edu.tw (P.-C.T.); koalbert@mail.cgu.edu.tw (A.M.-S.K.); yulinchen36@gmail.com (Y.-L.C.); 2 Healthy Aging Research Center, Chang Gung University, Taoyuan City 33302, Taiwan; 3 Molecular Infectious Disease Research Center, Chang Gung Memorial Hospital, Taoyuan City 33305, Taiwan; 4 Cardiovascular Department, Chang Gung Memorial Hospital, Taoyuan City 33305, Taiwan; 5 Division of Pediatric Infectious Diseases, Department of Pediatrics, Chang Gung Memorial Hospital, Taoyuan City 33305, Taiwan; 6 School of Medicine, Chang Gung University, Taoyuan City 33302, Taiwan; 7 Department of Surgery, College of Medicine, Kaohsiung Medical University, Kaohsiung City 80708, Taiwan; 8 Division of Cardiovascular Surgery, Department of Surgery, Kaohsiung Medical University Hospital, Kaohsiung City 80708, Taiwan

**Keywords:** atrial fibrillation, exosomal miRNA, sinus rhythm restoration, DESeq2, DIABLO model, early recurrence, biomarkers, rhythm control treatment

## Abstract

We aimed to identify serum exosomal microRNAs (miRNAs) associated with the transition from atrial fibrillation (AF) to sinus rhythm (SR) and investigate their potential as biomarkers for the early recurrence of AF within three months post-treatment. We collected blood samples from eight AF patients at Chang Gung Memorial Hospital in Taiwan both immediately before and within 14 days following rhythm control treatment. Exosomes were isolated from these samples, and small RNA sequencing was performed. Using DESeq2 analysis, we identified nine miRNAs (16-2-3p, 22-3p, 23a-3p, 23b-3p, 125a-5p, 328-3p, 423-5p, 504-5p, and 582-3p) associated with restoration to SR. Further analysis using the DIABLO model revealed a correlation between the decreased expression of miR-125a-5p and miR-328-3p and the early recurrence of AF. Furthermore, early recurrence is associated with a longer duration of AF, presumably indicating a more extensive state of underlying cardiac remodeling. In addition, the reads were mapped to mRNA sequences, leading to the identification of 14 mRNAs (*AC005041.1*, *ARHGEF12*, *AMT*, *ANO8*, *BCL11A*, *DIO3OS*, *EIF4ENIF1*, *G2E3-AS1*, *HERC3*, *LARS*, *NT5E*, *PITX1*, *SLC16A12*, and *ZBTB21*) associated with restoration to SR. Monitoring these serum exosomal miRNA and mRNA expression patterns may be beneficial for optimizing treatment outcomes in AF patients.

## 1. Introduction

Exosomes are small vesicles secreted by most cells [1]. They enclose cargo molecules, such as mRNA and miRNA. Exosomal miRNAs are small noncoding RNAs that regulate protein-coding genes. Unlike conventional miRNAs, which are found within cells or circulate freely, exosomal miRNAs are encapsulated within a double-membrane structure [1]. This encapsulation process, known as endocytosis, involves the inward folding of the cell’s plasma membrane to form a vesicle containing the miRNA. This provides them with high stability and resistance to degradation, making them ideal biomarkers in circulating fluids such as blood and saliva [1]. They can transport specific miRNAs from donor cells to recipient cells, influencing their function. Due to their high stability, specificity, and cell-targeting ability, exosomes have garnered considerable interest in cardiovascular disease research [2].

Atrial fibrillation (AF) is a heart condition characterized by rapid and irregular beating of the heart’s atrial chambers. This irregular rhythm may cause serious symptoms and complications such as blood clots, strokes, or heart failure [2]. The choice of treatment-rate control or rhythm control-depends on the patient’s symptoms and the severity of the AF [3]. Rate control treatment aims to regulate heart rate but not necessarily restoring normal sinus rhythm (SR). This treatment includes mostly of beta-blockers, calcium channel blockers, and digoxin. These medications slow the electrical conduction between the atria and the ventricles, lowering the heart rate. This enables them to alleviate symptoms such as palpitations, fatigue, and shortness of breath, improving the patient’s quality of life. Rhythm control treatment, on the other hand, seeks to restore and maintain normal SR. These include antiarrhythmic drug therapy, which regulates the electrical activity of the heart [4]; electrical cardioversion, which uses an electric shock to reset the heart’s rhythm [5]; and radiofrequency catheter ablation, a procedure that applies heat to destroy the area of the heart causing the irregular rhythm [6].

While miRNAs have been widely studied in AF [7], few studies have looked at exosomal miRNAs in AF [8,9]. A comprehensive list of the changes, such as upregulation or downregulation, in exosomal miRNAs involved with the transition from AF to SR is still needed. We propose that changes in the serum levels of certain exosomal miRNAs can indicate SR restoration. We will test this hypothesis by performing a longitudinal study in which miRNA levels are measured before and after rhythm control treatment.

These results may provide insight into the important regulators in treatment-refractory AF patients and the early recurrence of AF, which is defined as recurrence within three months after initiating rhythm control treatment. Understanding these changes may lead to improved treatment strategies for AF patients.

## 2. Results

### 2.1. Patient Characteristics

Table 1 shows the characteristics of the eight patients involved in this study. The patients were mostly male (75%), with ages ranging from 39 to 74 years and a median age of 63.5 years. All patients had been diagnosed with AF and were on rate control medications to manage their symptoms. None of the patients had previously undergone any rhythm control treatment. The AF duration, ranging from four months to six years, with a median of two years, represents the time from diagnosis to their first rhythm control treatment. Five patients had a long AF duration, defined as AF persisting for 12 months or more [10].

The left ventricular ejection fraction (LVEF), an indicator of heart function, varied among patients from 50% to 78%, with a median of 59%. Given that normal LVEF range of 55% to 70%, all of the patients had largely normal LVEFs. Each patient provided two blood samples: one immediately prior to treatment and another 1 to 14 days post-treatment, with a median collection time of 5 days. The timing of the second sample collection varied for practical reasons, such as pre-collection fasting for six hours, patients being discharged after the procedure, or patients returning to the clinic the following week.

Among the patients receiving rhythm control treatment, three underwent electrical cardioversion, while the remaining five were treated with radiofrequency catheter ablation. None of the patients had a history of coronary artery disease, atherosclerosis, or malignancy prior to treatment. However, two patients had diabetes, and the majority (*n* = 7) had hypertension. In the two-year follow-up period post-treatment, two patients experienced early recurrence within three months of treatment, while others had late recurrence, occurring more than one year post-treatment.

### 2.2. Differential Expression of Exosomal miRNAs and mRNAs

**Figure 1:**
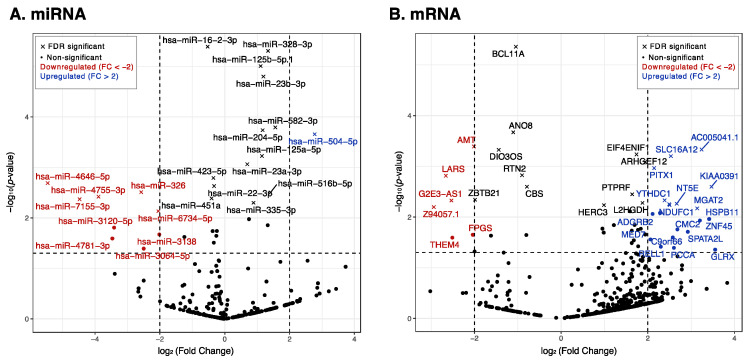
Volcano plots of 375 differentially expressed miRNAs and 743 mRNAs. Vertical dashed lines indicate a log 2-fold change with cutoffs set at −2 and 2. Horizontal dashed lines indicate a negative log 10 *p*-value with a cutoff set at 1.3 (*p*-value < 0.05). Cross symbols represent miRNAs and mRNAs that passed the BH-adjusted *p*-value, < 0.05, controlling for a false discovery rate (FDR).

**Figure 2:**
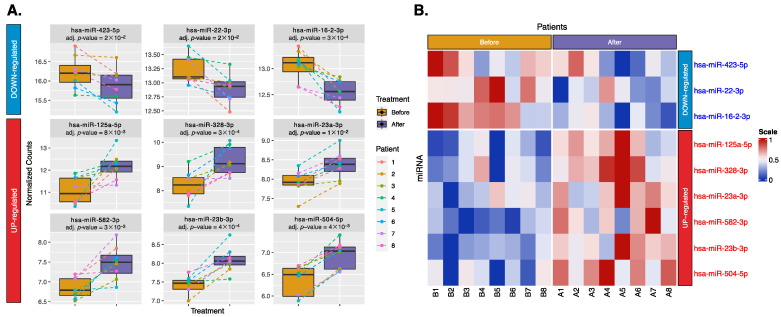
Nine significant exosomal miRNAs have a consistent treatment direction across all patients. (**A**) Boxplots show differences in expression levels between treatments, with colored dashed lines linking the expression levels of the same patient before (yellow) and after (purple) treatment. (**B**) Heatmap shows upregulation (red) or downregulation (blue), with the color scale reflecting z-score scaling between 0 and 1. Labels B1–B8 indicate patients 1–8 before treatment, and A1–A8 indicate patients 1–8 after treatment.

A total of 1061 miRNAs were mapped. After data cleaning, 375 miRNAs remained for analysis. Using DESeq2 in a paired design, we identified treatment-related changes while adjusting for individual differences. Figure 1A shows a volcano plot of these 375 miRNAs. Among these 375 miRNAs, 30 miRNAs had a *p*-value < 0.05, of which 9 miRNAs passed the Benjamini–Hochberg (BH)-adjusted *p*-value < 0.05, and indicated a consistent treatment direction. These nine miRNAs, including three downregulated (miR-423-5p, miR-22-3p, and miR-16-2-3p) and six upregulated (miR-125a-5p, miR-328-3p, miR-23a-3p, miR-582-3p, miR-23b-3p, and miR-504-5p) miRNAs, are shown in boxplots and a heatmap (Figure 2). These miRNAs may play a role in the restoration to normal SR and could serve as biomarkers for successful treatment.

**Figure 3:**
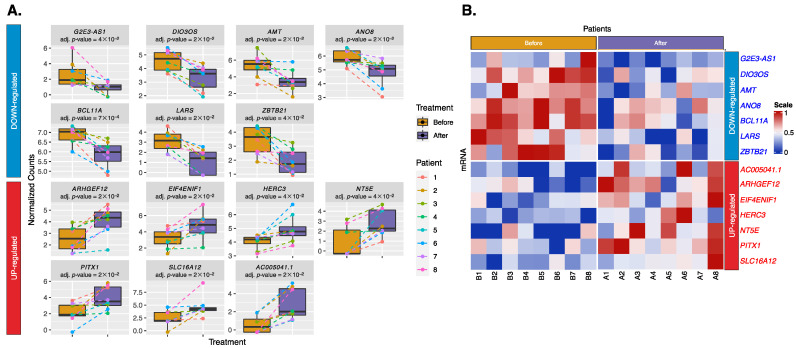
Fourteen significant exosomal mRNAs have a consistent treatment direction across all patients. (**A**) Boxplots show differences in expression levels between treatments, with colored dashed lines linking the expression levels of the same patient before (yellow) and after (purple) treatment. (**B**) Heatmap shows upregulation (red) or downregulation (blue), with the color scale reflecting z-score scaling between 0 and 1. Labels B1-B8 indicate patients 1-8 before treatment, and A1-A8 indicate patients 1-8 after treatment.

We retained 25,851 mRNAs that mapped to antisense RNA, lincRNA, and protein-coding RNA, all of which have previously been linked to AF. After data cleaning, 743 mRNAs remained. These were analyzed using DESeq2, and the results are shown in a volcano plot (Figure 1B). Among these 743 mRNAs, 51 mRNAs had a *p*-value < 0.05, of which 14 mRNAs passed the BH-adjusted *p*-value < 0.05 and indicated a consistent treatment direction. These 14 mRNAs, including 7 downregulated (*G2E3-AS1*, *DIO3OS*, *AMT*, *ANO8*, *BCL11A*, *LARS*, and *ZBTB21*) and 7 upregulated (*AC005041.1*, *ARHGEF12*, *EIF4ENIF1*, *HERC3*, *NT5E*, *PITX1*, and *SLC16A12*) mRNAs, are shown in boxplots and a heatmap (Figure 3).

### 2.3. Data Transformation and DIABLO Model

**Figure 4:**
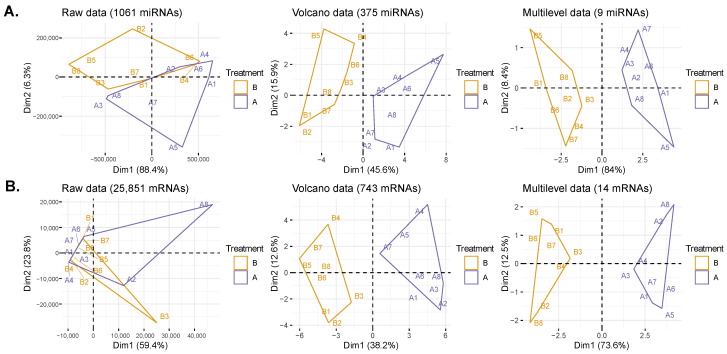
PCA shows a distinct separation between the clusters representing the before and after treatment groups. For both (**A**) miRNA and (**B**) mRNA, the PCA is performed at three stages of data filtering, depicted from left to right: raw data, data used for volcano plot, and data used for multilevel analysis. The labels B (orange) and A (purple) indicates before and after treatment, respectively, within these groups individual patients are labeled from 1 to 8.

Principal Component Analysis (PCA) was used to visualize patient clustering at each stage of the data transformation (Figure 4). The initial raw un-normalized data had overlaps, especially in the mRNA data. As we increasingly filtered for more significant miRNAs/mRNAs, the overlaps disappeared, and the two treatment clusters (before and after) became more distinct and separated, with the first principal component (PC1) explaining 74% to 84% of the total variance. This indicates that our final selection of nine miRNAs and fourteen mRNAs highly reflects the changes due to treatment and the restoration of normal SR.

The multilevel data (Figure 4) are a data transformation that enables the analysis of repeated measurements on the same patient in a DIABLO model, which can enhance the model’s classification accuracy. DIABLO was used to integrate phenotype–mRNA–miRNA datasets into a single analysis and to identify correlations between clinical phenotypes, mRNAs, and miRNAs. The AUROC (area under receiver operating characteristics) values of the final model per block (phenotype = 1; mRNA = 0.68; and miRNA = 0.7) indicate reasonable discriminative ability.

**Figure 5:**
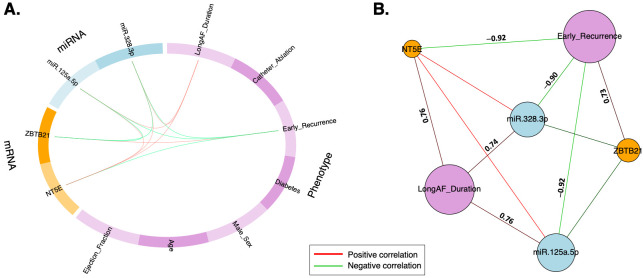
Correlations among multi-omic layers (7 clinical phenotypes, 14 miRNAs, 9 mRNAs) in predicting the early recurrence of AF post-treatment. These correlations are considered strong at a correlation coefficient above 0.7. (**A**) Circos plot shows that the early recurrence is negatively correlated with the expression levels of miR-125a-5p, miR-328-3p, and *NT5B*, and positively correlated with *ZBTB21*. (**B**) Relevance network plot shows the magnitude of each correlation, which is indicated by the numerical values on the lines connecting the variables.

Next, setting a correlation coefficient greater than 0.7, early recurrence can be correlated with miRNAs/mRNAs in a circos plot (Figure 5A). The relevance network plot provides further information, revealing that the early recurrence of AF is negatively correlated with miR-125a-5p, miR-328-3p, and *NT5E* but positively correlated with *ZBTB21*. The long AF duration phenotype also remained in the network. However, it seems to have an indirect connection with early recurrence via the three aforementioned markers. It showed a positive correlation with these markers but no relationship with *ZBTB21* (Figure 5). This points to the complex interplay between these markers, AF duration, and recurrence.

## 3. Discussion

We examined the changes in serum exosomal expression levels of miRNAs and mRNAs during the transition from AF to SR. We identified nine miRNAs (16-2-3p, 22-3p, 23a-3p, 23b-3p, 125a-5p, 328-3p, 423-5p, 504-5p, and 582-3p) and fourteen mRNAs (*AC005041.1*, *ARHGEF12*, *AMT*, *ANO8*, *BCL11A*, *DIO3OS*, *EIF4ENIF1*, *G2E3-AS1*, *HERC3*, *LARS*, *NT5E*, *PITX1*, *SLC16A12*, and *ZBTB21*). A predictive model that maximizes the covariance between all pairs of datasets (phenotype, mRNA, and miRNA) reveals that, as the expression levels of miR-125a-5p, miR-328-3p, and *NT5E* decrease, the likelihood of the early recurrence of AF after treatment increases.

### 3.1. Part 1: The Role of miRNAs in SR Restoration

Most of our findings with miRNA changes seem to be linked to cardiac remodeling (fibrosis or inflammation), while some are related to the electrical activity of the heart. Their detailed descriptions are as follows.

#### 3.1.1. Downregulated miRNAs:

*miR-16-2-3p* has been previously suggested as a biomarker for AF [11]; this miRNA is thought to be involved in cardiac remodeling after acute myocardial infarction [12] and regulates fatty acid degradation in endothelial cells, a process that could improve coronary microvascular dysfunction [13]. Its downregulation after treatment supports its significance as a biomarker for AF and may reflect a decrease in cardiac remodeling activity, which is related to the progression of AF. *miR-22-3p* is highly expressed in cardiac striated muscle tissue and is assumed to be essential for normal cardiac remodeling in response to stress [14]. Its downregulation in our study suggests that the heart recovers from the cardiac stress of AF and reverts to a more normal state. *miR-423-5p* reduces the phosphorylation of calcium-handling proteins in AF [15]. Its downregulation suggests that the electrical properties of the heart return to normal, promoting the restoration of SR.

#### 3.1.2. Upregulated miRNAs:

*miR-23a-3p* and *miR-23b-3p* have been linked to cardiac fibrosis [16,17] and myocardial dysfunction [18,19], both of which are important components of the cardiac remodeling process. Their enhanced expression may contribute to the restoration of SR by lessening fibrosis and improving the elasticity and contractility of the heart muscle, facilitating the restoration of normal heart rhythm. *miR-125a-5p* is upregulated in response to treatment. Our model correlates a decrease in its expression to the early recurrence of AF, a finding that aligns with three previous studies. These studies found that AF recurrence is linked with a reduction in the expression of 125a-5p following ablation by targeting the pro-inflammatory factor IL-6R [20]. A longitudinal study conducted over a 12-month period found that AF recurrence is associated with levels of 125a-5p being threefold lower after ablation compared with pre-ablation [21]. The third study, similar to ours, specifically examined exosomal miRNAs [9]. These findings suggest that miR-125a-5p may play an important role in the inflammatory response associated with AF and that its upregulation could indicate reduced inflammation and improved heart function.

*miR-328-3p* targets the *CACNA1C* and *CACNB1* genes, which produce the α1c- and β1 subunits of the cardiac L-type Ca^2+^ channel [22,23]. Its upregulation following treatment may influence the electrical properties of the atria. However, our model correlates its decreased expression to the early recurrence of AF, implying a reversal of this process. This suggests that the positive benefits of upregulation may be short-lived and that sustained upregulation may be necessary to prevent AF recurrence. *miR-504-5p* is downregulated during cardiomyopathy, which may affect heart rhythm [24]. Its upregulation after treatment may be attributable to a reduction in the pathological processes associated with cardiomyopathy, such as myocardial fibrosis and hypertrophy. *miR-582-3p* regulates endothelial miRNA expression via *NRF2* in response to proatherogenic stimuli and is implicated in the apoptosis of vascular smooth muscle cells [25]. Its upregulation after treatment may indicate a reduction in proatherogenic stimuli and an improvement in vascular function, contributing to the restoration of SR.

### 3.2. Part 2: The Role of mRNAs in SR Restoration

The functions of the mRNAs we found are diverse, ranging from protein synthesis and metabolism to cellular signaling, and some are directly connected to AF risk. Their descriptions are below.

#### 3.2.1. Downregulated mRNAs:

*G2E3* is related to ubiquitin E3 ligases and may contribute to heart failure due to hyper-ubiquitylation and ubiquitin–proteasome system impairment [26]. Its downregulation might indicate improved cardiac remodeling and a decrease in heart failure symptoms. *DIO3OS* has been linked to thyroid function, anemia [27], and the remodeling of coronary artery plaques in atherosclerosis [28]. Its downregulation may be associated with a decrease in coronary artery risk. *AMT* encodes an enzyme involved in glycine metabolism [29]. Its downregulation may affect protein synthesis and metabolic processes, suggesting a change in metabolic activity. *ANO8* is implicated in calcium signaling [30]. Its downregulation might impact cellular signaling pathways and homeostasis, suggesting a return to normal calcium signaling in the heart. *BCL11A* is related to the exercise-induced heart rate response [31]. Its downregulation may impact cardiovascular responses and adaptations to physical activity, showing improved exercise tolerance. *LARS* regulates protein synthesis, especially the attachment of leucine to tRNA, and suppresses osteosarcoma cells [32]. Its downregulation might influence protein synthesis and metabolic processes. *ZBTB21* has been linked to hypertension in the Uyghur people [33]. Its downregulation may indicate a mechanism for blood pressure regulation and a reduction in hypertension risk.

#### 3.2.2. Upregulated mRNAs:

*AC005041.1*’s function is not well documented in the literature. *ARHGEF12* is related to AF [34]. Its upregulation might indicate a mechanism for the progression of AF and an increased risk of AF. *EIF4ENIF1* has been linked to primary ovarian insufficiency [35]. *HERC3* encodes a protein that acts as a ubiquitin–protein ligase. Its upregulation might indicate changes in protein degradation or signaling pathways. *NT5E*, also known as *CD73*, is an enzyme involved in the purinergic signaling pathway [36] and has been linked to heart failure [37]. Its upregulation may indicate an increased risk of heart failure. *PITX1* is related to the heritability of AF [38]. Its upregulation may indicate an increased risk of AF. *SLC16A12* is related to AF [39]. Its upregulation may indicate an increased risk of AF.

### 3.3. Part 3: Early Recurrence of AF and Underlying Cardiac Remodeling

The DIABLO model predicts a strong correlation (with a coefficient greater than 0.7) between reduced levels of miR-125a-5p, miR-328-3p, and *NT5E* and early AF recurrence. This is despite the overall upregulation of these markers in all patients following treatment. The complex relationship between these markers and AF recurrence may be attributed to the heart not having fully adapted to the restored SR. This could indicate an incomplete or unsuccessful reversal of the remodeling process. Even though the heart rhythm has been restored to normal, the underlying molecular changes associated with long-term AF and cardiac remodeling might still persist, making the heart more susceptible to AF recurrence.

Maladaptive myocardial remodeling involves a variety of changes, such as cardiac hypertrophy, cardiomyocyte injury, cardiac fibrosis, angiogenesis, and inflammatory response [40]. Atrial fibrosis, in particular, disrupts normal electrical conduction, creating a substrate for local re-entry and contributing to the progression of AF. For instance, miR-125a-5p [41] and miR-328-3p [42] have been linked to cardiac hypertrophy and fibrosis, two key aspects of cardiac remodeling. *NT5E* has been linked to regulatory networks in cardiac remodeling and heart failure [37].

We observed that early recurrence is associated with a prolonged AF duration; i.e., the course of AF lasted for 12 months or longer. The longer the heart remains in a state of AF, the more extensive the remodeling of the heart tissue becomes. This remodeling involves changes in the size and shape of the heart chambers, as well as alterations in the cellular and molecular properties of the heart tissue. A previous study showed that patients with a long AF duration and early recurrence are independently associated with late recurrence [10]. This suggests that individuals who experience AF shortly after treatment may have more severe or persistent forms of the disease, making them more likely to experience AF again at a later point in time.

Furthermore, the inverse relationship between early recurrence and long AF duration via the three markers may suggest that they are involved in processes that counteract the progression of AF, such as anti-inflammatory or anti-fibrotic pathways. When these markers are highly expressed (which is associated with a long AF duration), these protective mechanisms might be more active, thereby reducing the likelihood of early recurrence. On the other hand, *ZBTB21*, which is positively correlated with early recurrence, does not show any correlation with long AF duration. As it is related to hypertension, it might be involved in different biological processes that are independent of the duration of AF but contribute to the early recurrence of AF.

### 3.4. Part 4: Strengths and Limitations of this Study

This study offers several strengths. Firstly, it focuses on serum exosomal miRNAs, which are more stable and convenient for clinical detection and monitoring than other tissues. Secondly, it examines two time points—before and after treatment—minimizing variability and confounding factors due to individual differences. Thirdly, it prioritizes finding true positives by imposing a 5% false discovery rate and a consistent treatment direction across all patients while sacrificing sensitivity due to long-term changes. Lastly, the implementation of DIABLO allows for a single analysis that takes into account multiple data types and their interactions.

However, our study also has limitations. Primarily, our analysis was performed on Taiwanese males, which may limit the generalizability and reproducibility of our findings to the broader population. Additionally, a small sample size might lead to overfitting in the prediction model. The timing of the second blood sample collection, which had a median time of five days, could affect the levels of exosomal miRNAs/mRNAs that degrade rapidly. Measuring before and after treatment within a limited timeframe restricts our analysis to the causal inference of these specific biomarkers in response to the treatment, potentially overlooking dynamic and longitudinal alterations in expression profiles. Lastly, our study was constrained by an incomplete mRNA library, as the small RNA-seq may not efficiently capture full-length mRNAs. Our results require experimental validation, as well as validation in a larger cohort of patients. These limitations should be carefully considered when interpreting our results.

In terms of implementing these findings in routine clinical practice for AF, several potential challenges and solutions should be considered. Factors such as the source of sample collection (blood, urine, or saliva) can influence the yield and composition of exosomes. Each method of isolation and purification (ultracentrifugation, chromatography, or immunoaffinity capture) can offer improved purity but may not be suitable for all sample types. Challenges also exist in miRNA and mRNA extraction and profiling and clinical validation in a diverse group of patients. These challenges are being addressed via the development of better solutions such as standardized protocols; next-generation sequencing technologies; and collaborations between researchers, clinicians, and regulatory bodies.

## 4. Materials and Methods

### 4.1. Participant Recruitment and Sample Collection

Participants were recruited from the LinKou Chang Gung Memorial Hospital in Taiwan between July 2018 and April 2019. The study enrolled patients diagnosed with AF who underwent rhythm control treatment, either electrical cardioversion or radiofrequency catheter ablation. Each patient was followed for at least two years, with the final patient being monitored until October 2021. Prior to participating in this study, all participants provided their written informed consent. The study protocol adhered to the ethical guidelines of the 1975 Declaration of Helsinki and was approved by the Institutional Review Board of Chang Gung Medical Foundation (IRB No: 201407002B0 and 201801478A3, approved on 20 December 2014 and 29 November 2018, respectively). Blood samples were collected from each participant before and after the procedure, with participants instructed to fast for a minimum of six hours before blood sample collection. Demographic and clinical data were gathered from medical records to establish the participants’ baseline characteristics.

### 4.2. RNA Purification

Total RNA was isolated within two hours of blood sample collection. The procedures for RNA purification, small RNA library construction, and sequencing have been previously described [43]. The RNA content, purity, and integrity were assessed using the RNA Nano 6000 Assay Kit of the Agilent Bioanalyzer 2100 System (Agilent Technologies, Santa Clara, CA, USA) prior to sequencing.

### 4.3. Small RNA Library Preparation and Sequencing

Illumina sequencing libraries were prepared using the NEXTflexTM small RNA-seq kit v3 Guide (Bioo Scientific, Austin, TX, USA; 5132-05) as per the manufacturer’s instructions. In each library, 60 nanograms of pure RNA was ligated to 3′ and 5′ adaptors. The RNA was then reverse-transcribed into complementary DNA (cDNA) using adaptor-specific primers. The miRNA library was purified and amplified using both universal and barcode-specific primers. The library was then size-selected and extracted from a 6% TBE-PAGE gel for miRNA dispersion. The yield and size distribution of the small RNA libraries were evaluated using the Agilent 2100 Bioanalyzer instrument and the Agilent Technologies High-Sensitivity DNA Assay. Each library was sequenced at the same concentration using an Illumina NextSeq 500 platform. Reads from the small RNA-seq experiment were also mapped to mRNA sequences.

### 4.4. Exosomal microRNA and mRNA Analysis

Data cleaning of the raw data included removing miRNAs and mRNAs with fewer than ten counts across all patients, as well as those that were inconsistent with the treatment direction. We used DESeq2 [18] and a paired design (~Treatment + Patient) to identify differentially expressed miRNA and mRNAs, accounting for individual differences. Top genes that met a Benjamini–Hochberg (BH)-adjusted *p*-value threshold of 0.05 and demonstrated consistent treatment direction across all patients were kept. DESeq2’s normalization function, varianceStabilizingTransformation (vst), was performed to the count data to visualize the findings using PCA, boxplots, and heatmaps.

### 4.5. DIABLO Analysis for Multiomics Integration and Prediction

To adjust for repeated measurements, we used the withinVariation function from the mixOmics package [44] on the vst normalized mRNA and miRNA data. DIABLO [45], a supervised multiblock sPLS-DA technique, was used to combine three datasets: 7 clinical phenotypes, 14 mRNAs, and 9 miRNAs. It generated latent components, which were combinations of the original variables from each layer, in a way that maximized the covariance between all pairs of datasets. These latent components were then used to build a predictive model for the early recurrence of AF post-treatment. The model differentiates between distinct outcome categories, such as recurrence versus no recurrence. We used all of the data to train the model. The design matrix was set to prioritize discriminative ability, and the results are shown as circos and relevance network plots.

### 4.6. Statistical Analysis

All visuals were created using the R software, version 4.3.2, and PCA was carried out with factoextra package in R. The *ggplot2* package was used to create volcano plots and boxplots. Heatmaps were made using ComplexHeatmap [46].

## 5. Conclusions

We have identified specific serum exosomal miRNAs and mRNAs that are associated with the transition from AF to SR. Particularly, a decrease in the expression of miR-125a-5p and miR-328-3p was found to correlate with the early recurrence of AF, suggesting these miRNAs could serve as potential biomarkers for predicting recurrence. Further research is needed to validate these findings and to explore their clinical implications in a larger cohort of patients.

## Figures and Tables

**Table 1:** Clinical characteristics of eight AF patients.

Patient	Sex	Age (Years)	AF Duration (Months)	Recurrence	Diabetes	LVEF (%)	Treatment Type
1	Female	65	9	Late	No	68	RFCA
2	Female	61	15	Late	No	65	EC
3	Male	39	33	Late	Yes	50	RFCA
4	Male	66	89	Late	No	53	EC
5	Male	70	35	Late	Yes	50	RFCA
6	Male	74	9	Early	No	78	EC
7	Male	56	5	Early	No	50	EC
8	Male	62	22	Late	No	65	EC

AF duration, time between diagnosis and treatment; recurrence, post-treatment recurrence of AF within three months (early) and more than three months (late); LVEF, left ventricular ejection fraction; RFCA, radiofrequency catheter ablation; EC, electrical cardioversion.

## Data Availability

The data presented in this study are available from the corresponding authors upon request.

## Abbreviations

AF	Atrial fibrillation
SR	Sinus rhythm
RFCA	Radiofrequency catheter ablation
EC	Electrical cardioversion
miRNA	microRNA
PCA	Principal component analysis
